# *Saccharomyces cerevisiae* Genes Involved in Survival of Heat Shock

**DOI:** 10.1534/g3.113.007971

**Published:** 2013-10-18

**Authors:** Stefanie Jarolim, Anita Ayer, Bethany Pillay, Allison C. Gee, Alex Phrakaysone, Gabriel G. Perrone, Michael Breitenbach, Ian W. Dawes

**Affiliations:** * Division of Genetics, Department of Cell Biology, University of Salzburg, Salzburg, A-5020 Austria; † School of Biotechnology and Biomolecular Sciences, University of New South Wales, Sydney, NSW 2052, Australia; ‡ School of Science and Health, University of Western Sydney, Penrith, NSW 1797, Australia

**Keywords:** heat shock, genome-wide screen, *Saccharomyces cerevisiae*, tryptophan metabolism, DNA repair

## Abstract

The heat-shock response in cells, involving increased transcription of a specific set of genes in response to a sudden increase in temperature, is a highly conserved biological response occurring in all organisms. Despite considerable attention to the processes activated during heat shock, less is known about the role of genes in survival of a sudden temperature increase. *Saccharomyces cerevisiae* genes involved in the maintenance of heat-shock resistance in exponential and stationary phase were identified by screening the homozygous diploid deletants in nonessential genes and the heterozygous diploid mutants in essential genes for survival after a sudden shift in temperature from 30 to 50°. More than a thousand genes were identified that led to altered sensitivity to heat shock, with little overlap between them and those previously identified to affect thermotolerance. There was also little overlap with genes that are activated or repressed during heat-shock, with only 5% of them regulated by the heat-shock transcription factor. The target of rapamycin and protein kinase A pathways, lipid metabolism, vacuolar H^+^-ATPase, vacuolar protein sorting, and mitochondrial genome maintenance/translation were critical to maintenance of resistance. Mutants affected in l-tryptophan metabolism were heat-shock resistant in both growth phases; those affected in cytoplasmic ribosome biogenesis and DNA double-strand break repair were resistant in stationary phase, and in mRNA catabolic processes in exponential phase. Mutations affecting mitochondrial genome maintenance were highly represented in sensitive mutants. The cell division transcription factor Swi6p and Hac1p involved in the unfolded protein response also play roles in maintenance of heat-shock resistance.

Heat stress affects a broad range of cellular processes, including transient arrest of cell division (Rowley *et al.* 1993), uncoupling of oxidative phosphorylation (Patriarca and Maresca 1990), accumulation of misfolded proteins (Vidair *et al.* 1996), damage to membranes and cytoskeletal structures, and defective trafficking through the secretory pathway (Richter *et al.* 2010). Heat stress is also thought to be associated with pronounced production of reactive oxygen species (Davidson and Schiestl 2001; Sakaki *et al.* 2003) whose identity and source *in vivo* have yet to be determined.

The heat-shock response in cells, involving the increased transcription of a specific set of genes in response to an increase in temperature, is highly conserved. First noted in *Drosophila* as a distinct puffing pattern induced on polytene chromosomes by temperature shock (Ritossa 1962), subsequent research in *Saccharomyces cerevisiae* showed that transcription of set of genes increased after heat shock (Miller *et al.* 1979) and the rate of synthesis of some proteins produced at significant levels under normal growth temperatures was reduced (McAlister *et al.* 1979). After heat shock, cells decrease general protein synthesis to focus on the synthesis of heat-shock proteins needed for repair and survival. These survival proteins have a broad range of functions, including molecular chaperones to assist with protein folding and disaggregation (Hendrick and Hartl 1993), cell wall remodeling (Imazu and Sakurai 2005), ubiquitin-related protein degradation, DNA- and RNA-modifying enzymes, metabolic enzymes, regulatory proteins, and others involved with maintenance of cell structure (Richter *et al.* 2010). The precise number and nature of genes induced by the heat-shock response may vary between organisms; however, at the fundamental level of chaperones, the response is conserved (Richter *et al.* 2010).

Despite the considerable attention given to heat-shock regulation (for a review, see Morano *et al.* 2012), less is known about the cellular functions that are required to maintain resistance to a sudden heat shock. There have been several transcriptomic analyses of how yeast cells respond to various forms of heat shock (Gasch *et al.* 2000; Causton *et al.* 2001; Segal *et al.* 2003; Matsumoto *et al.* 2005), and a genome-wide analysis of gene promoters that bind the heat-shock factor (Hahn *et al.* 2004). However, major differences exist between genes whose expression is altered in response to a given stress *vs.* those needed for resistance to the stress (Giaever *et al.* 2002; Thorpe *et al.* 2004; Berry and Gasch, 2008; Mitchell *et al.* 2009). Genome-wide screens have identified mutants affected in thermotolerance (the ability to grow at increased temperatures; Auesukaree *et al.* 2009) and acquired thermotolerance (the ability of cells to survive an usually nonpermissive temperature due to exposure to a milder heat or other stress; Mir *et al.* 2009). There are, however, significant differences between acute heat shock and thermotolerance (De Virgilio *et al.* 1991; Lindquist and Kim 1996). Teng *et al.* (2011) used a ramped heat stimulus screen of the yeast deletion collection as an approach to identify strains that have increased survival of a death stimulus. This involved delivery of the heat stimulus over a period of 20 min to stationary phase cells. Here we report the results of screening of the genome-wide deletion mutants in *S. cerevisiae* for resistance and sensitivity to a sudden heat shock and the identification of functions that are critical for heat-shock survival in both exponential and stationary phase cells.

## Methods and Materials

### *S. cerevisiae* strains and growth conditions

BY4743 homozygous diploid and BY4741 haploid deletants for all nonessential genes from the *Saccharomyces* Gene Deletion Project were obtained from EUROpean *Saccharomyces Cerevisiae* ARchive for Functional Analysis (EUROSCARF) (Winzeler *et al.* 1999). The heterozygous essential gene deletion set in the BY4743 background was obtained from Open Biosystems (now Thermo Fisher Scientific Inc.). The wild-type BY4743 has the genotype *MAT***a***/MATa his3Δ1/his3Δ1 leu2Δ0/leu2Δ0 met15Δ0/MET15LYS2/lysΔ0 ura3Δ0/ura3Δ0*; BY4741 is *MAT***a** *his3Δ1 leu2Δ0 met15Δ0 ura3Δ0*. Homozygous diploids were used for screening to minimize where possible the effect of any secondary mutations in the haploids used to generate the diploids. The BY4741 haploid was used for tryptophan starvation and cycloheximide inhibition experiments. To assess the contribution of essential genes in survival of heat shock, ~1100 BY4743 heterozygous diploid deletion mutants (Open Biosystems) were used. Yeast extract peptone dextrose (YEPD) medium contained 2% (w/v) glucose, 2% (w/v) bactopeptone, and 1% yeast extract, and synthetic minimal dextrose (SD) medium contained 2% (w/v) glucose, 0.17% yeast nitrogen base (Difco), 0.5% ammonium sulfate (Oxoid), and auxotrophic requirements at 40 mg/L where necessary. Media were solidified by adding 2% (w/v) agar. All cells were incubated at 28°.

### Heat-shock assay

Microtitre plates with cell suspensions in each well were placed in a water bath at the appropriate temperature such that the plate was immersed to the level of the medium in the plate. At 0, 30, 60, 90, 120, 180, 240, 300, and 360 min, samples were taken with a 96-well replicator and transferred to a YEPD agar plate (140 cm diameter), which was incubated at 30°. Growth was scored visually after 1, 2, and 3 d, either directly, or from photographs, by comparison with the growth of the control strain BY4743. The absence of growth or highly reduced colony number was indicative of sensitive strains and better survival/growth than the wild-type strain was scored as resistant.

For screening of the nonessential homozygous diploid collection, strains in 96-well microtitre plates were thawed from the mutant collection and incubated for 3 d in YEPD. Plates were reinoculated to another microtitre plate containing YEPD medium as a master plate and grown for 3 d at 28°. From these master plates, a sample was replicated with a 96-well replicator to a microtitre plate containing YEPD and grown for 3 d. Strains in liquid culture were used for the heat-shock treatment of cells in stationary phase. Simultaneously, a second sample was replicated from the master plate onto a 140-cm YEPD agar plate and grown at 28° for 3 d. After 3 d, colonies from the agar plate were replicated with a 96-well replicator to a prewarmed microtitre plate containing YEPD, incubated for 6 hr at 28°, and used for the heat-shock assay of cells in exponential phase. The optical density of a sample measured at a wavelength of 600 nm, or OD_600_, was measured with a microplate reader (SpectraMax 340) at the beginning and end of the 6-hr cultivation period to determine the extent of growth of the mutants.

Mutants that were scored as sensitive or resistant were transferred to a new microtitre plate and retested with the same procedure. Rescreening was performed four times to eliminate as many false-positive results as possible.

### Heat-shock screening of the essential heterozygous diploid collection

For analysis of exponential phase heat-shock survival for essential genes, strains were inoculated from stationary phase YEPD cultures into 150 μL of fresh YEPD medium liquid, grown for 17 hr and subjected to heat shock as per the methodology outlined for non-essential genes.

### Spot tests for effects of L-tryptophan starvation on heat-shock survival

The requirement for l-tryptophan for the homozygous *trp5* derivative of BY4743 was determined by inoculating cells in SD medium containing l-tryptophan in the range of 0−100 μM, allowing growth for 2 d at 30° and determining the final OD_600_. From this, it was determined that cells would be starved for l-tryptophan if grown in 10 μM l-tryptophan and that concentrations greater than 15 μM would be in excess. Heat-shock sensitivity for cells was determined via spot tests. Cultures of the wild-type and *trp5* mutant were pregrown in SD medium with different concentrations of l-tryptophan at 30° in 24-well plates (1 mL per well, shaking on a reciprocal shaker at 700 rpm). At an appropriate OD_600_, 300 μL of culture was transferred to a preheated Eppendorf tube and shaken at 600 rpm in an Eppendorf (Thermomixer Comfort) heating block at 50°. Aliquots were taken at 30-min intervals and diluted to OD_600_ of 1-, 0.3-, 0.1-, 0.03-, 0.01-, and 5-μL samples at each dilution spotted on a YEPD plate which was then incubated at 30° for 2 d.

### Amino acid analyses

Amino acids in cells were assayed with high-performance liquid chromatography (HPLC) of amino acids derivatized using Pico Tag chemistry (Waters Corporation, Milford, MA). Cells from 50 mL of culture were harvested on a wet 0.44-μm glass fiber filter and washed with 50 mL of ice-cold SD medium without added amino acids. The prefilter was extracted with 30 mL of cold 5% (w/v) perchloric acid for 5 min with occasional agitation. The lysate was neutralized with cold 5 M potassium hydroxide and centrifuged to remove the precipitate. A 2.2-mL sample of each lysate was centrifuged at 13,000*g* for 5 min and 2 mL of supernatant was transferred to a 4-mL glass vial, stored at −80° overnight, and subsequently freeze dried at −50°. Dried samples were resuspended in 200 μL of internal standard solution (0.2 M methionine sulfone in 0.1 M HCl) and 50 μL transferred to sampling tubes (6 mm × 50 mm) for drying in a centrifugal vacuum concentrator. Samples were then resuspended in 10 μL of drying solution (2:2:1 methanol:1 M sodium acetate:TEA), followed by another round of drying. Derivatization reagent (7:1:1:1 methanol:TEA:water:PITC; 20 μL) was added to samples with thorough mixing, and then samples were incubated at room temperature for 20 min without light. Samples were dried again, resuspended in 100 μL of Pico-TAG sample diluent (HPLC grade)m and 40 μL was then transferred to sampling vials. Samples were analyzed within 24 hr by HPLC on 3.9-mm × 15-cm Pico-TAG Reverse Phase Free Amino Acids columns at 46° with flow rate 1 mL/min and UV detection at 254 nm. Data were analyzed using Agilent Chemstation software.

### Data analysis

Overrepresented molecular functions, biological processes, and cellular component categories were determined using Funspec (Robinson *et al.* 2002) and YeastMine (Balakrishnan *et al.* 2012). Gene-specific data were obtained from the Saccharomyces Genome Database (www.yeastgenome.org/; Cherry *et al.* 2012). Transcription factor binding data were obtained using the YEASTRACT programs (Abdulrehman *et al.* 2011) and gene expression data from published microarray experiments were obtained using the SPELL program (Hibbs *et al.* 2007).

## Results and Discussion

### Development and validation of screening approach

Systemic screening of the *S. cerevisiae* deletion set required development of a method to handle the number of strains involved and to ensure reproducible results. The optimum temperature at which to discriminate both sensitive and resistant strains was assessed using the control strain BY4743 and the sensitive homozygous diploid *hsp104Δ* mutant. *HSP104* is expressed at low levels at normal temperatures, is induced very strongly by heat, and its product is critical for cell survival after heat shock (Lindquist and Kim 1996).

Flat-bottom 96-well microtitre plates were inoculated with wild-type cells, except for a row that contained the otherwise isogenic *hsp104Δ/hsp104Δ* diploid. Cells were grown to exponential phase and stationary phase (3 d) and the plates exposed to a range of temperatures from 42° to 55°. The optimal temperature was 50°, at which loss of viability of the *hsp104Δ* strain was much faster than the wild type; at lower temperatures the wild type survived for more than 2 hr without a significant decrease in viability; at 55°, death was too rapid to distinguish the wild type from the sensitive strain. Although stationary-phase cells showed a better survival than exponential cells at 50°, it was still possible to distinguish sensitive mutants. No difference was observed for cells that were heat shocked in the medium in which they had grown or those transferred to either fresh YEPD or dilute (1:10) YEPD. YEPD medium was chosen for the screen because it allowed growth of a large proportion of auxotrophic mutants.

### Identification of heat-shock−resistant and −sensitive mutants

**Figure 1:**
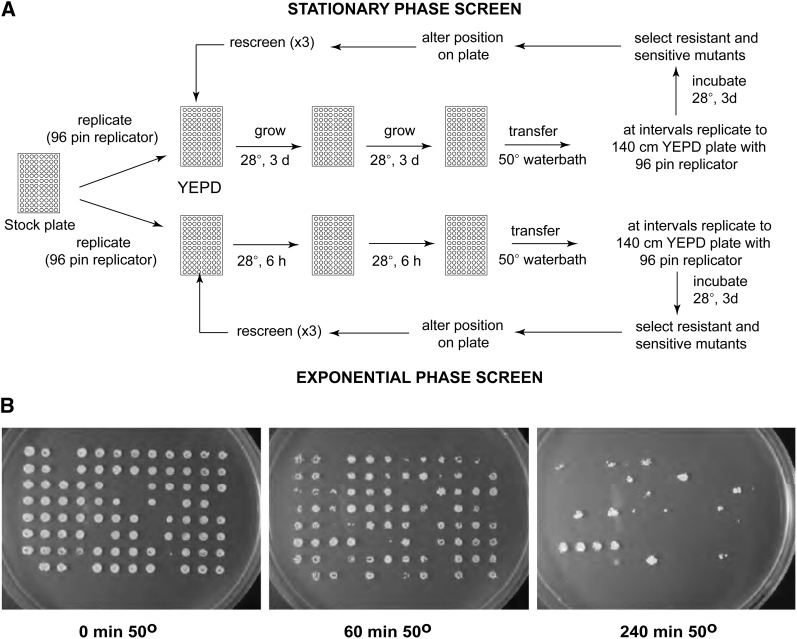
Screening of the nonessential deletion mutant collection. (A) Schematic diagram to illustrate the screening protocol. Mutants were grown in YEPD in 96-well plates as described in the *Materials and Methods* and after heat treatment at 50° for the times indicated, the wells were replicated to YEPD plates with a 96-pin replicator. (B) Data for plate 2000-40 from the EUROSCARF collection, with cells grown to stationary phase.

For stationary phase cultures, freshly thawed strains from the EUROSCARF strain collection were inoculated in YEPD medium using a 96-pin replicator and grown for 3 d. These strains were reinoculated into YEPD medium in 96-well plates and grown to stationary phase for 3 d to ensure the cells were in the postdiauxic phase and assayed for heat-shock survival. A second batch was pregrown on YEPD to exponential phase, replicated with a 96-pin replicator to fresh prewarmed YEPD, and incubated for 6 hr at 30° before assay. No correlation was observed between heat-shock sensitivity and cell growth rate. Figure 1A gives an outline of the screening protocol, and results for a representative plate from this screening are shown in Figure 1B. A slight edge effect was noted with cells at the edge of the plates showing better survival than those in the middle. Therefore, the resistant and sensitive mutants from the first screen were re-located on the plates for subsequent screening. Each mutant from the preliminary screening was retested three times to minimize false–positive results, with little change in the number of strains observed between the third and fourth screen.

**Figure 2:**
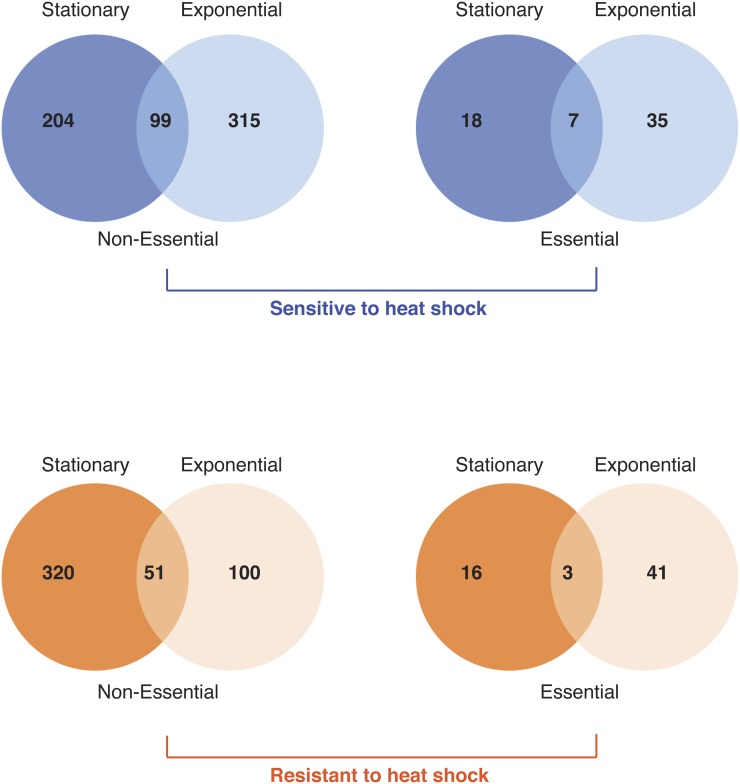
Overlap between sets of heat-shock−sensitive and −resistant mutants identified from stationary- and exponential-phase screens of the nonessential and heterozygous essential mutant collections.

From the stationary phase screens a total of 320 (16) resistant and 204 (18) sensitive mutants were identified, whereas from exponential phase there were 100 (41) resistant and 315 (35) sensitive strains; the number in parentheses indicates the number of mutants identified in the essential gene set. These data are provided in Supporting Information, File S1. Resistant strains were classified from 1 to 4 (increasing resistance) and sensitive strains from −1 to −4 (increasing sensitivity) on the basis of survival time. Overall, 1049 resistant and sensitive mutants were identified, with only 51(3) mutants identified as resistant and 99 (7) mutants identified as sensitive in both the exponential- and stationary-phase screens (Figure 2), indicating that there are major differences between the cells in these two different phases in the genes (and to some extent functions) that are important for heat-shock survival. Sixteen mutants were resistant in stationary phase and sensitive in exponential phase.

### Genes required for heat-shock resistance differ from those needed for acquired thermotolerance

The aforementioned data were compared with those obtained in two genome-wide screens for mutants defective in thermotolerance. The first screen, for acquired thermotolerance, identified only 37 mutants that were less able to adapt to a 50° heat shock after pretreatment with heat at 37° (Mir *et al.* 2009), far fewer than those identified in the present study, in part because the authors excluded any strain that was slow-growing in the control (*i.e.*, following pretreatment to 37° without subsequent heat shock). Only 12 of those mutants were identified in the present study, and only seven of these (*hsp104Δ*, *rpl8AΔ*, *rpl37BΔ*, *spt3Δ*, *fzo1Δ*, *atp17Δ*, and *mnn11Δ*) were sensitive to heat shock; the others were resistant. The probability of this overlap being enrichment beyond chance was *P* = 0.1 from the hypergeometric test. The second screen, for intrinsic thermotolerance, identified 178 mutants that showed reduced growth on YEPD plates at 37° (Auesukaree *et al.* 2009). Of those mutants, only 43 were found in the present screen, which is not significant relative to chance (*P* = 2.9). However 25 thermosensitive mutants did show a significant overlap (*P* = 6 × 10^−6^) with the exponential phase heat-shock sensitive mutants indicating that there are some functions in common between thermotolerance and heat-shock resistance during exponential growth. Interestingly eight thermosensitive mutants were heat-shock resistant. Of the main functions represented by the mutants identified by (Auesukaree *et al.* 2009), vacuolar sorting and transcriptional regulation were common to those found in the present study, but few specific genes affecting transcriptional components were common to both sets of data.

These data indicate that there is not a strong overlap in the genes that are required for acquired/intrinsic thermotolerance and those needed for resistance to heat shock, and that the processes differ considerably in terms of the cellular functions that are important for cell survival. This may reflect that cells are resistant to greater temperatures following a brief treatment rather than under prolonged exposure, and possibly to a greater reliance on established (constitutive) rather than inducible functions to survive sudden heat shock. Berry and Gasch (2008) have shown that gene expression changes triggered by a single dose of stress are not required to survive that stimulus but rather serve a protective role against future stress, which provides an explanation of the lack of extensive overlap between the data presented here and those for acquired thermotolerance (Mir *et al.* 2009). There was also little overlap between deletion mutants affected in heat shock and those involved in cell death as identified by Tang *et al.* (2011) (See Table S1).

All genes whose deletions conferred resistance or sensitivity were analyzed for their transcriptional response to heat stress, using data from Gasch *et al.* (2000). Those data were obtained from two different heat-shock protocols with cells shifted over several different temperature ranges, and the results varied according to the protocol used. There was, however, only ~30% overall correspondence between the gene deletions that affect heat-shock resistance/sensitivity and the transcriptional changes in response to heat shock (see Table S2 and File S2). Despite the relatively large number of genes in the overlap, this was not significant relative to chance (*P* = 0.64) due to the large number of genes identified in both screens. Moreover, a search of the genes from each of the sets of deletion mutants for heat-shock factor (Hsf1p) binding to their promoters using the YEASTRACT database indicated that only a low percentage of genes in any of the mutant categories bind Hsf1p (exponential sensitive: 6%; stationary sensitive: 8%; exponential resistant: 8%; stationary resistant: 8%), each of which represents a relatively low percentage (in every case <5%) of all the genes that bind Hsf1p. A similar situation was found when analyzing the data for binding of the general stress transcription factors, with none of the separate data sets including more than 6% of genes regulated by Msn2p or Msn4p. This finding is consistent with the situation for other stresses (Giaever *et al.* 2002; Schüller *et al.* 2004; Thorpe *et al.* 2004) and further indicates that many of the genes needed to survive the sudden imposition of a stress need to be expressed constitutively so that their products are in place when the stress is applied.

### Tryptophan metabolism is critical to maintenance of heat shock in both exponential and stationary phase

**Figure 3:**
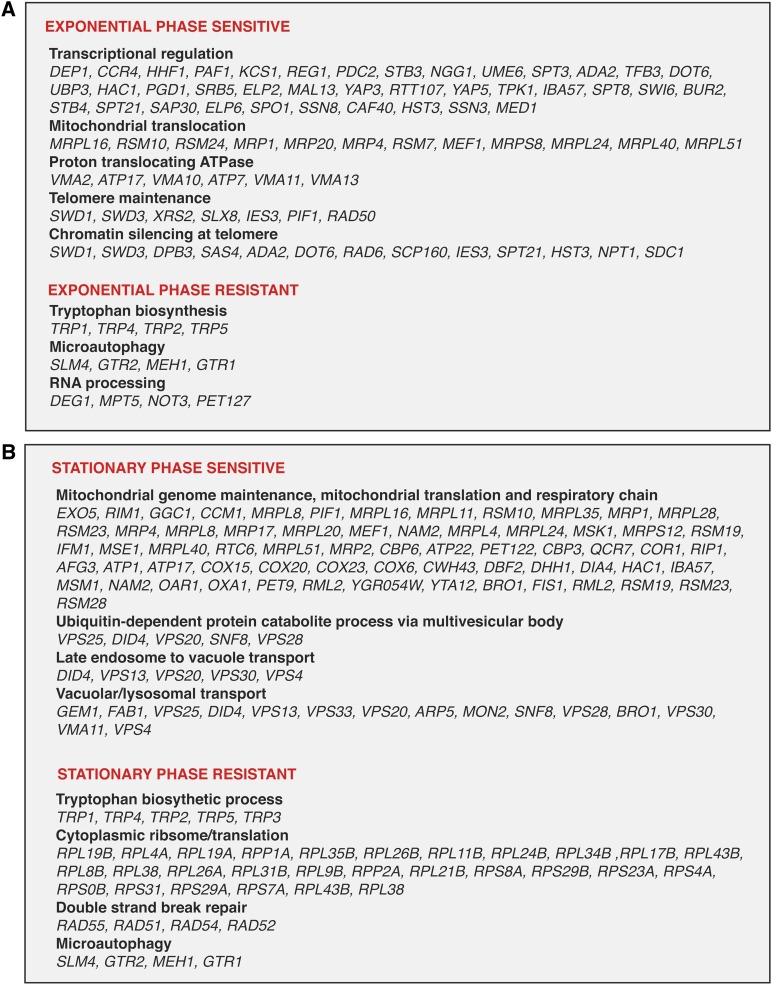
Overrepresented biological processes in the heat-shock mutant data for (A) exponential-phase cells and (B) stationary-phase cells according to the FunSpec program (Robinson *et al.* 2002)

**Figure 4:**
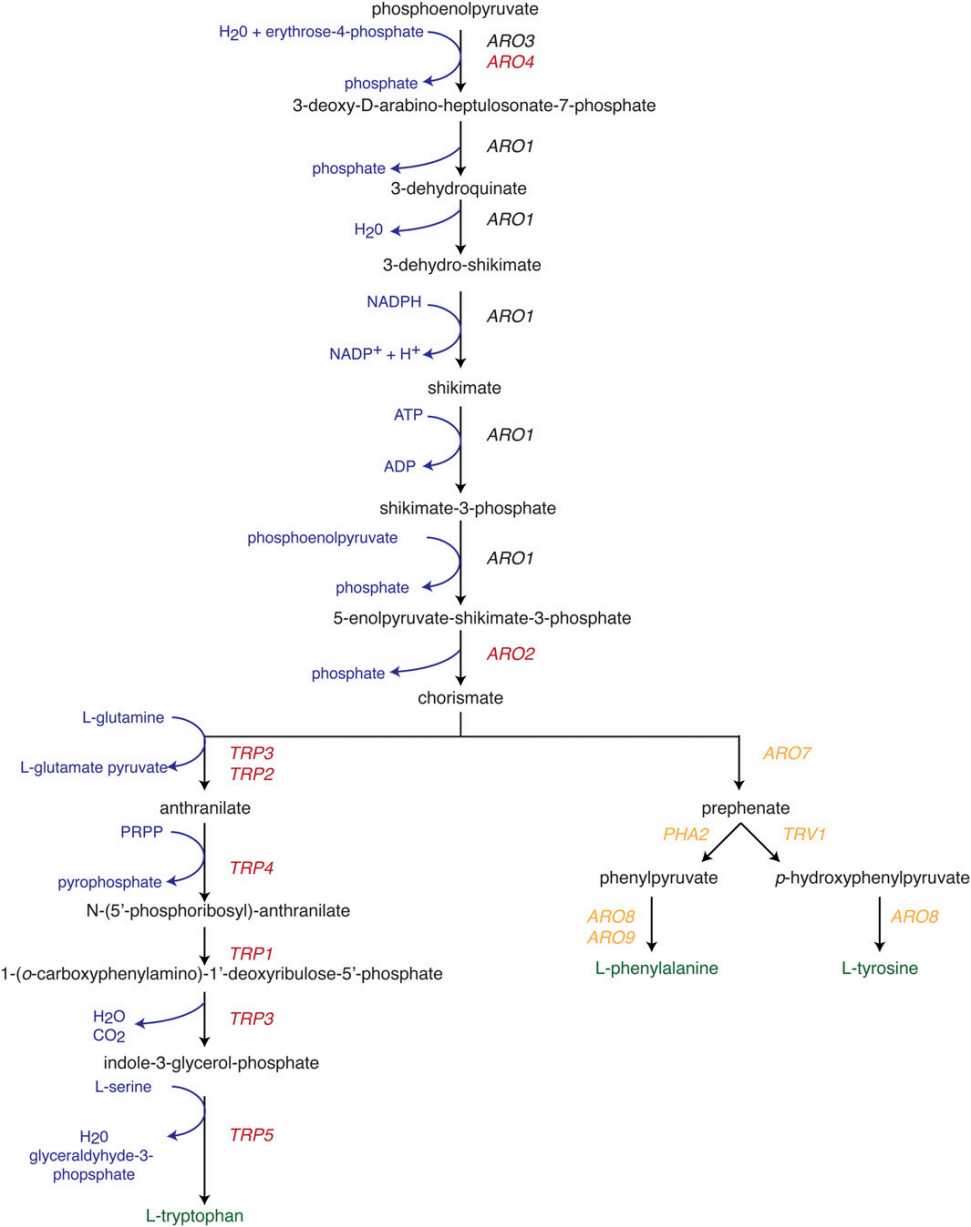
Mutations affecting aromatic amino acid biosynthesis lead to heat-shock resistance in both exponential and stationary phase. Pathway modified from the Saccharomyces Genome Database. Genes that when deleted led to heat-shock resistance are indicated in red.

To identify cell functions important in maintaining resistance and sensitivity, genes were grouped into overrepresented molecular functions, biological processes, and cellular component categories (Figure 3, A and B) according to FunSpec and Yeastmine programs. A striking feature of mutants resistant to heat shock in both exponential and stationary phase was the involvement of aromatic amino acid biosynthesis (Figure 4), one of the most significantly enriched functional categories of any gene set examined and *trpΔ* mutants were among the more resistant mutants identified. Mutations affecting synthesis of the tryptophan precursors erythrose-4-phosphate (*tkl1Δ*) and 5-phosphoribosyl-1-pyrophosphate (*prs3Δ*) were also resistant. This effect appeared to be specific to l-tryptophan and not L-phenylalanine or L-tyrosine since only the tryptophan branch of the pathway was represented in the resistant mutants.

**Figure 5:**
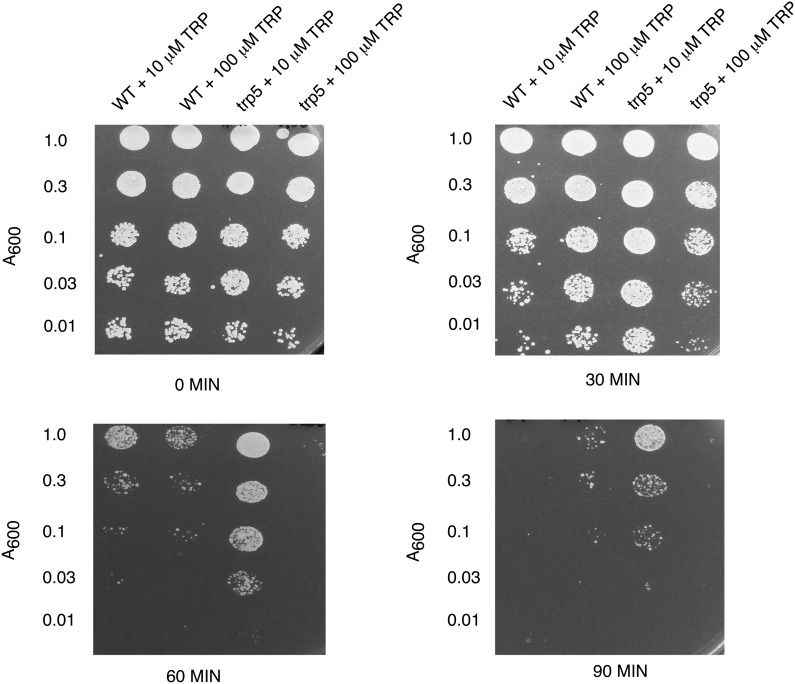
Tryptophan starvation induces the heat-shock response. Cells of the BY4741 wild-type and *trp5* mutant were grown overnight at 30° in SD medium containing auxotrophic requirements and 10 μM l-tryptophan (which leads to starvation for tryptophan) or 100 μM l-tryptophan (which is in excess). Heat-shock resistance for each culture was determined as indicated in the *Materials and Methods*.

To verify that l-tryptophan depletion affects heat-shock survival, the heat resistances of the *trp5Δ* mutant and wild type were determined for cells grown to starvation in defined (SD) medium with a range of concentrations of added l-tryptophan. In cells starved for tryptophan, there was a substantial increase in heat-shock resistance; when tryptophan was present in excess, there was no difference between the mutant and the wild type (Figure 5). This finding raises the question of why the *trp* deletants are heat-shock resistant in YEPD medium. One possible explanation is that tryptophan is heat labile and present in relatively low concentrations in YEPD, and hence the mutants were probably depleted for tryptophan under the conditions used in the screen. Amino acid analysis of YEPD medium used indicated that the concentration of free tryptophan was very low (<125 nM; 26 μg/mL). One possibility is that under tryptophan limitation the cells may activate the target of rapamycin (TOR) pathway, which is a critical event in the heat-shock response as indicated below.

### Mutations affecting cytoplasmic ribosome biogenesis and DNA double-strand break repair lead to heat-shock resistance in stationary phase cells

**Figure 6:**
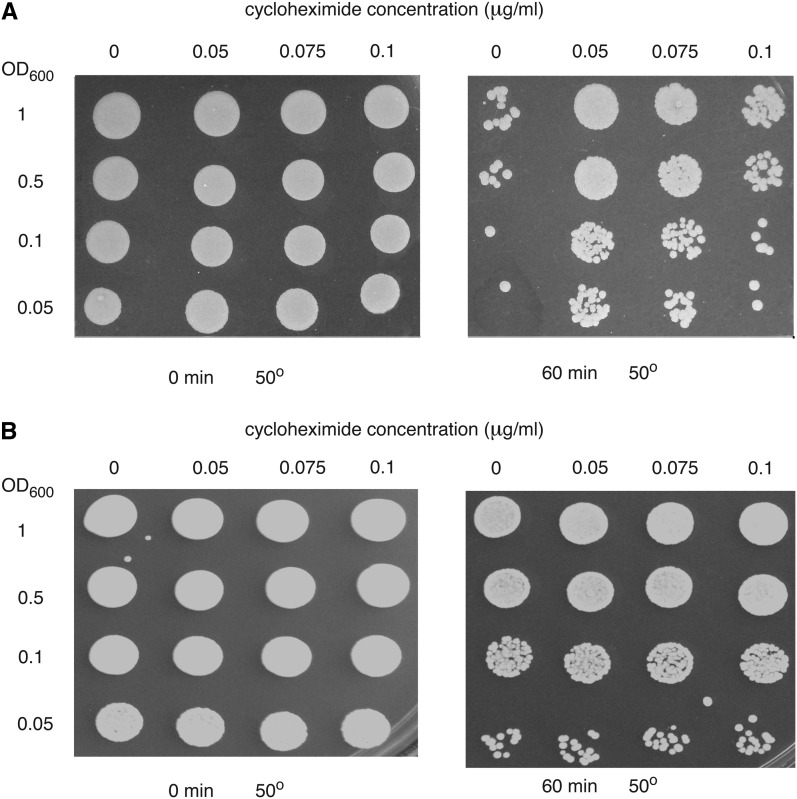
Partial inhibition of protein synthesis by low concentrations of cycloheximide leads to strong heat-shock resistance. Cells of haploid strain BY4741 were grown to mid-log phase (A) and stationary phase (B) in SD medium with auxotrophic requirements and then treated with the indicated concentrations of cycloheximide at 30° for 60 min. One aliquot was serially diluted to the OD_600_ indicated and spotted on YEPD plates. The other aliquot was treated at 50° for 60 min prior to dilution and spotting. In exponential phase cycloheximide at 0.1 μg/mL led to a 50% reduction in the growth rate of the cells but did not affect the final growth yield.

A large proportion of mutants that were resistant in stationary phase were those affected in cytoplasmic ribosome biogenesis and translation (Figure 3, A and B). Many of these mutants (especially those encoding ribosomal proteins) are viable because there is another functional homologous gene encoded in the genome, which indicates that a reduction in capacity for protein synthesis, especially in stationary phase, may lead to an up-regulation of systems providing heat-shock resistance. In many cases, deletion of only one of the two homologs encoding a ribosomal protein led to resistance. This could indicate that one gene of a homologous pair has a greater role in translation (especially during starvation) than the other, is regulated differently, that the homologs have different functions in cells that have been heat stressed, or that deletion of one of the two homologs leads to a reduced rate of protein synthesis and this stimulates mechanisms enhancing heat resistance. It is unlikely that regulation of expression is the basis for this difference since available transcription data in the Serial Pattern of *Expression* Levels Locator (SPELL; spell2.princeton.edu/) database show a high degree of coregulation of homologous pairs of ribosomal genes for cells in exponential and stationary phases as well as during heat shock. It is interesting to note that slowing the rate of protein synthesis in exponential phase cells without completely inhibiting it, using very low concentrations of cycloheximide (0.05 µg/mL), also led to strong heat-shock resistance (Figure 6A). Because treatment with cycloheximide is considered to lead to chemical stress, this result is not necessarily conclusive despite the very low concentrations used. However, there was no effect of cycloheximide on heat resistance of the wild type in stationary phase when the rate of translation is low, but chemical stress should still operate (Figure 6B).

Mutants affected in recombinational repair of DNA double-strand breaks during mitosis and meiosis belonging to the *RAD52* epistasis group (*rad 50Δ*, *rad51Δ*, *rad52Δ*, *rad54Δ*, *rdh54Δ*, and *rad55Δ*) were also resistant to heat shock in stationary phase but not in exponential phase. These mutations make cells sensitive to methylating agents as well as those causing strand breakage. This implies that there may be cross-talk between DNA repair and heat-shock resistance such that in stationary phase the function of the DNA repair pathway lowers the cells’ ability to resist heat shock, or in the mutants the cells adapt to become more heat-shock resistant in response to an accumulation of DNA damage in stationary phase cells. It has been reported that DNA damage can in some way signal through the TOR pathway – as a determinant of cell survival in response to DNA damage (Shen *et al.* 2007). Because activation of the TOR pathway is a critical event in the heat-shock response (see the section *Role of the TOR and protein kinase A pathways and the EGO and GSE complexes*), it may provide the mechanism that connects the DNA damsage response to heat shock, and some explanation why the DNA repair mutants were only sensitive in stationary phase, because the TOR pathway also responds to the cell’s nutritional status.

### Role of the TOR and protein kinase A pathways and the EGO and GSE complexes

The EGO and GSE complexes were highly overrepresented (in the cellular component category) in both exponential and stationary phase heat-resistant mutants (*slm4Δ*, *gtr2Δ*, *meh1Δ*, and *gtr1Δ*). The EGO complex plays a critical role in activation of microautophagy by counterbalancing the influx of membranous material toward the vacuolar membrane leading to efflux of vacuolar material via microautophagy rather than the retrograde pathway (Dubouloz *et al.* 2005). Gtr2p acts in a vacuolar membrane-associated protein complex with Ego1p and Ego3p to ensure proper exit from rapamycin-induced growth arrest, implicating some aspect of the TOR pathway and nutritional sensing. Large-scale genetic analyses of the EGO complex confirm the existence of a growth control mechanism originating at the vacuolar membrane. Although genes involved in autophagic processes were identified in the screen (*ATG4*, *ATG17*, *ATG20*) and autophagy is important to heat-shock resistance, no mutants, resistant or sensitive, that were affected in functions specific to microautophagy were found, and hence microautophagy *per se* is possibly not involved in maintenance of resistance to heat shock.

The related GSE complex (GTPase-containing complex for Gap1p sorting in the endosomes) is required for proper sorting of Gap1p from the late endosome for eventual delivery to the plasma membrane. The complex contains two small GTPases (Gtr1p and Gtr2p) and three other proteins (Ybr077cp, Ykr007wp, and Ltv1p) that are located in the late endosomal membrane (Gao and Kaiser 2006). These authors postulated that the complex functions in regulating the TOR pathway possibly by re-establishing a balance in the distribution of membranes in the entire endo membranous system. One possible explanation of these data are that heat damages the membrane in such a way that the operation of EGO and GSE systems affecting membrane trafficking, and aspects of nutrient sensing, are deleterious to the cell’s recovery without the involvement of the subsequent steps of microautophagy. Inhibition of membrane sorting, or TOR signaling may assist cells to recover if membrane proteins have been damaged.

The involvement of the TOR pathway in heat-stress resistance/sensitivity has been described (Bandhakavi *et al.* 2008) and is highlighted by the data in the present screen because the *rim15Δ* mutant was sensitive in both exponential and stationary phase and *tco89Δ* was resistant in stationary phase cells. Rim15p is involved in transmission of TOR pathway signaling to the Msn2p/4p transcription factors (Roosen *et al.* 2005), and Tco89p is a component of the TOR complex (Reinke *et al.* 2004). A range of functions associated with cAMP and protein kinase A signaling came up in the exponential heat-shock screen, including Ira1p (GTPase-activating protein that negatively regulates RAS), Cyr1p (adenylate cyclase), Cdc25p (guanine nucleotide exchange factor that regulates adenylate cyclase through activation of Ras1p and Ras2p), and Tpk1p (one of three protein kinase A catalytic subunits). These data are in accord with the view that Rim15p responds to both TOR (positively) and PKA (negatively) in a heat-shock as well as nutrient-sensing pathway leading to activation of the Msn2p transcription factor (Roosen *et al.* 2005). Moreover, deletion of *NPR2*, whose product regulates TORC1 activity on amino acid starvation, led to sensitivity in exponential phase.

Further indication of the critical role of the TOR pathway and the heat-shock response comes from identification of the *ccr4Δ* and *not3Δ* mutants. The CCR4-NOT complex is a conserved global regulator of gene expression, which senses and/or transmits nutrient and stress signals to various effectors. The effectors in yeast of this complex are considered to be the general transcription factor TFIID, and Msn2p, regulating expression of genes regulated by stress-responsive elements (STRE) (Lenssen *et al.* 2005). *NOT3* has also been predicted on the basis of extensive microarray data to be involved in the transcriptional response to heat shock (Segal *et al.* 2003). Caf40p is a component of the CCR4-NOT complex (along with Cdc39p, Caf1p, Dbf2p, and Caf130p; Chen *et al.* 2001); the *caf40Δ* mutant was sensitive in exponential phase and *dbf2Δ* was sensitive in stationary phase. Lenssen *et al.* (2005) have shown that the constitutively high level of STRE-driven expression in *ccr4-not* mutants results from two independent effects. Loss of CCR4-NOT function causes a Msn2-independent redistribution of TFIID on promoters with a bias for STRE-controlled over ribosomal protein gene promoters. In parallel, loss of CCR4-NOT complex function results in a change in the post-translational modification of Msn2, which depends on the type 1 protein phosphatase Glc7 and its subunit Bud14. The *glc7Δ* mutant (in the essential gene screen) was resistant in stationary phase and the *glc8Δ* mutant was sensitive in stationary phase to heat shock (*GLC8* encodes the regulatory subunit of Glc7p).

### mRNA catabolic processes affect heat-shock resistance in exponential phase

Mutants resistant in exponential phase were enriched for functions involved in mRNA catabolism, including deadenylation-dependent decay (*ccr4Δ*, *puf3Δ*, *puf4Δ*, and *mpt5Δ*); “nuclear-transcribed mRNA catabolism” (*lsm2Δ*, *mrt4Δ*, *puf3Δ*, and *vts1Δ*); and poly(A)-tail shortening (*ccr4Δ*, *not3Δ*, and *vts1Δ*). The main effect of the CCR4/NOT complex may be related to its effects on expression of genes with a STRE element in the promoter rather than with deadenylation of transcripts. However, deadenylation via the CCR4/NOT complex also may be involved in heat-shock sensitivity because *MPT5* encodes a protein that binds to the 3′-untranslated region of specific mRNAs, including those involved in cell-wall integrity, chronological lifespan, and chromatin modification, and recruits the CCR4-NOT deadenylase complex to mRNAs to promote deadenylation and, along with Dhh1p and Dcp1p, decapping and decay. Puf3p and Pet127p are involved in mitochondrial functions, whereas Puf4p binds mRNAs encoding nucleolar ribosomal RNA-processing factors and Mrt4p localizes to the nucleolus and is involved in mRNA turnover and ribosome assembly.

### Transcriptional regulation in the maintenance of heat-shock resistance: involvement of cell division, unfolded protein response, and telomere function

One of the more significant functional categories for the heat-shock−sensitive mutants in exponential phase was transcription and transcriptional control (Figure 3). The mutants covered a diverse range of functions and include components of the basic transcriptional and chromatin modifying machinery as well as some involved in transcript processing. A number of these genes are involved in telomere maintenance or silencing of telomeric genes, and telomere silencing was one of the most significant gene functions identified for exponential phase sensitive mutants.

Interestingly, haplo-insufficiency of *HSF1* (*HSF1*/*hsf1Δ*) led to sensitivity to heat shock in exponential phase, but not stationary phase, and as indicated previously, only a relatively low proportion of the genes implicated in heat-shock sensitivity (when the gene is deleted) have Hsf1p bound to the promoter. The general stress activators (Msn2p or Msn4p) were not represented in either phase probably due to the redundancy of their function. Heat stress in secretory proteins, or overexpression of wild-type secretory proteins, cause unfolded proteins to accumulate in the endoplasmic reticulum (ER), triggering the unfolded protein response (UPR) mediated by the Hac1p transcription factor (Kaufman 1999). The role of the UPR is indicated by the sensitivity of the *hac1Δ* mutant.

The list of transcription factors includes many with roles in the mediator complex and chromatin remodeling/silencing, as well as the SAGA histone acetyltransferase complex (represented by the *ada2Δ*, *ngg1Δ*, *spt3Δ*, and *spt8Δ* mutants), which is involved in transcriptional activation of UPR genes and other stress genes, including those regulated by Hsf1p and Msn2/4p.

Maintenance of optimal heat-shock resistance clearly requires interplay of systems/functions dependent on many specific transcription factors. One particularly interesting set of these factors included those involved in cell-cycle progression at the G1-S phase boundary. These include Swi6p, which is a component of both the MBF and SBF G1-specific transcription factors, Msa1p (an activator of MBF and SBF), and Smi1p, which is involved in regulating cell-wall synthesis and proposed to be involved in coordinating cell-cycle progression with cell-wall integrity. Loss of any of these three proteins led to heat-shock sensitivity in exponential phase and would delay progression into S phase. Heat shock causes cell-cycle arrest in G1 phase accompanied by decreased expression of the G1 cyclins, Cln1p and Cln2p (Rowley *et al.* 1993). This decreased expression of *CLN1* and *CLN2* was reported to be independent of Swi4p (Rowley *et al.* 1993), which associates with Swi6p to form the SBF factor. Because expression of the two cyclin genes is regulated by both SBF and MBF (Breeden 1996; Ferrezuelo *et al.* 2010), the observed effect of deleting Swi6p, or its activator Msa1p, indicates that Swi6p is involved in some way in the heat-shock response and it could be acting through expression of the cyclin genes. It was, however, not possible to discern a wider role of the cell cycle in heat-shock resistance. Of the 800 mutants reported to be affected in cell division (Sherlock *et al.* 2001), 41 were affected in heat shock in exponential phase, and 49 in stationary phase; among these, no cell-cycle stage was overrepresented relative to other stages.

Iron metabolism is implicated in heat-shock resistance from the Yap5p iron-sensing transcription factor that regulates iron storage in the vacuole and possibly Msn1p, which is involved in iron uptake (along with a range of other processes including regulation of invertase and glucoamylase expression). Other transcription factors whose deletion led to a lower degree of sensitivity included Dep1p, involved in regulation of structural phospholipid biosynthesis genes and metabolically unrelated genes, as well as maintenance of telomeres; Mks1p (pleiotropic negative transcriptional regulator involved in Ras-cAMP and lysine biosynthetic pathways, nitrogen regulation and retrograde mitochondria-to-nucleus signaling); and Gcr2p (transcriptional activator of genes involved in glycolysis).

Gene redundancy would possibly affect the range of functions that might be detected by the mutant screening, and analysis of functions regulated by the transcription factors identified in the screen might reveal ones that are missed by virtue of redundancy. As an example, we have identified the functions of genes that bind Hac1p (using Yeastrac and Funspec databases). Apart from the obvious ER-associated and protein-folding functions, this transcription factor also regulates genes involved in protein targeting and sorting in the ER, those encoding the peroxiredoxins Tsa1p and Tsa2p, and genes involved in ATP synthase activity. Of these, the peroxiredoxins are functionally redundant, and relevant to oxidative stress, which is discussed below (see the section *Heat shock and reactive oxygen species*).

### Mitochondrial functions, especially genome maintenance, protein synthesis and respiratory chain function, are important for the maintenance of intrinsic heat resistance

Mutations affecting mitochondrial genome maintenance and translation were highly represented in sensitive mutants in either growth phase. Because the mitochondrial genome encodes components of the respiratory chain and the F_0_F_1_-ATPase, a functional respiratory chain is clearly important for heat-shock resistance, and this is highlighted by the many sensitive mutants that were deleted for components of the respiratory chain complexes, ubiquinone biosynthesis or functions needed for assembly of the complexes (Figure 3). This finding is in strong contrast with the role of the cytoplasmic ribosome discussed earlier. The mutants were screened on YEPD medium in which cells generate ATP mainly via fermentation rather than respiration; hence, these data indicate an important role for mitochondrial respiration even under conditions in which mitochondrial respiration or other mitochondrial functions are not essential.

### The vacuolar H^+^-ATPase (V-ATPase) and vacuolar protein sorting are critical for resistance

**Figure 7:**
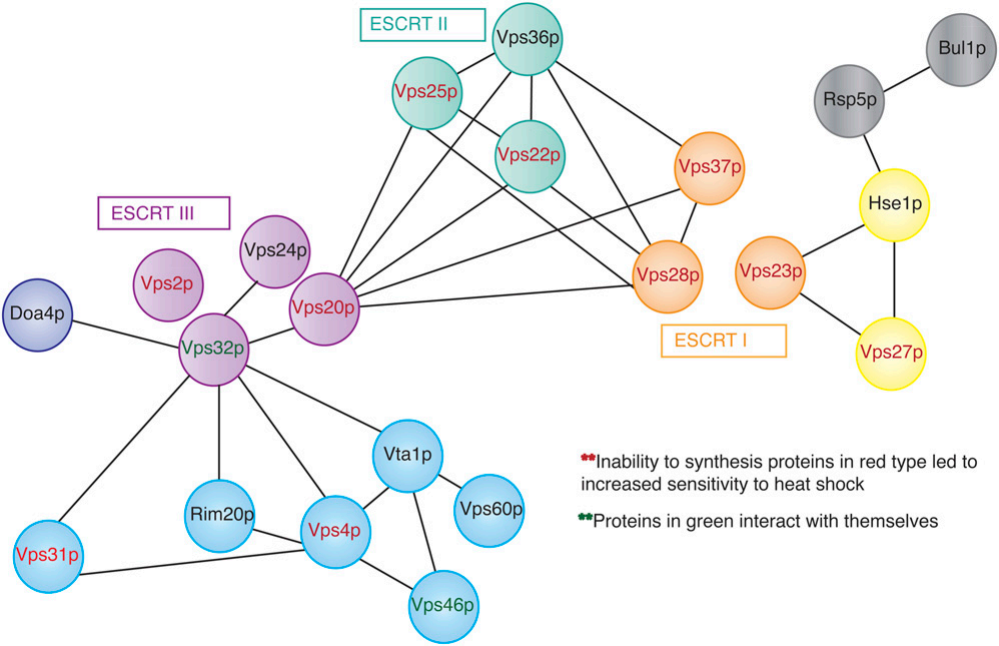
ESCRT complex functions involved in maintenance of heat-shock resistance. Proteins that when missing led to heat-shock sensitivity are indicated in red text. The interrelationships between ESCRT functions are described in (Bowers *et al.* 2004) from which the diagram is adapted.

A number of heat-shock sensitive mutants indicated the importance of the vacuole and an intact endosomal sorting pathway in the heat-shock response. There was strong overlap between genes involved in vacuolar protein sorting (VPS) identified in a genome-wide screen for VPS genes (Bonangelino *et al.* 2002) and those that are important for heat-shock resistance. The main vacuolar functions required for resistance during exponential growth are those involved in late endosome function, lysosomal and vacuolar degradation, and vacuolar transport. In stationary phase the sensitive mutants also represented vacuolar functions involved in protein sorting, protein fate and complex assembly. Mutants defective in the vacuolar H^+^-ATPase (*vma2Δ*, *vma3Δ*, and *vma21Δ*) were among the most heat-shock sensitive. Mutants defective in VPS were mainly sensitive; *vps4Δ*, *vps20Δ*, *vps25Δ*, *vps27Δ*, and *vps28Δ* are class E VPS mutants that accumulate an exaggerated form of a prevacuolar compartment in which the 60-kDa vacuolar H^+^-ATPase subunit is sequestered separate from the vacuole (Raymond *et al.* 1992). Certain class E VPS gene products are proposed to form three distinct endosomal sorting complexes (ESCRT-I, II, III), which are required for sorting proteins to the luminal membranes of multivesicular bodies. In *S. cerevisiae* deficiencies in the ESCRT machinery trigger the mistargeting of endocytic and biosynthetic ubiquitinated cargoes to the limiting membrane of the vacuole. Figure 7 illustrates the interaction of the ESCRT complexes (Bowers *et al.* 2004) and their overlap with proteins that when deleted led to heat-shock sensitivity.

Vacuolar acidification is important for many processes, including endocytosis, targeting of newly synthesized lysosomal enzymes, and other molecular targeting processes (Stevens and Forgac 1997). However, in conjunction with the Pma1p plasma membrane H^+^-transporter, the V-ATPase complex is also important for maintenance of normal pH homeostasis in yeast cells (Martinez-Munoz and Kane 2008). Heat shock has been found to lead to a substantial decrease (~1 pH unit) in cytoplasmic pH (Ayer *et al.* 2013) which perturbs redox regulation in the cytoplasm and which would stress the cellular capacity to maintain pH homeostasis in the absence of the vacuolar V-ATPase.

### Heat shock and reactive oxygen species

Deletion of *superoxide dismutase* (*SOD)1* (encoding the Cu/Zn superoxide dismutase located mainly in the cytoplasm, but also in the mitochondrial inner membrane space; Sturtz *et al.* 2001) led to sensitivity in stationary phase when cells would have respiratory activity. Oxidative stress originating from superoxide anion radicals (O_2_**·**^−^) produced in the mitochondrial respiratory chain has long been considered to play a large role in the toxicity of heat stress, and overexpression of SOD has been shown to increase yeast viability during heat stress (Davidson *et al.* 1996). Overexpression of *SOD2* also has been shown to extend chronological lifespan but reduces replicative lifespan (Harris *et al.* 2003; Fabrizio *et al.* 2004). However, there was no indication in the screen data that there is a need to remove reactive oxygen species other than O_2_**·**^−^ as part of the initial response to heat shock because neither of the catalase mutants, nor any of the mutants deleted for glutathione reductase, glutathione peroxidases, or thioredoxins, were identified and, significantly, deletion of the Yap1p transcription factor regulating these systems did not affect heat-shock resistance. Although gene redundancy may affect the outcome of a screen, it should be noted that mutants affected in single catalase or other antioxidant activities do show an oxidative stress phenotype. It therefore appears that “oxidative stress” during heat shock is restricted to the need to remove superoxide rather than other reactive oxygen species such as H_2_O_2_ or other peroxides. There was, however, significant overlap (*P* < 0.001) with the present data sets and those found in screening five different compounds leading to the generation of reactive oxygen species in cells (menadione, hydrogen peroxide, cumene hydroperoxide, diamide, and linoleic acid hydroperoxide; Thorpe *et al.* 2004). The overlap of the datasets was 153 genes comparing the exponential phase screen with the overall oxidative stress screen, and 162 if the stationary phase screen was compared. However, within the overlapping gene sets, none of the oxidants was represented more often than any other.

### Role of lipid metabolism

Sphingolipid long-chain bases (LCBs) have been implicated in thermotolerance and also the heat-shock response (Dickson 1998; Skrzypek *et al.* 1999). *S. cerevisiae* cells contain two types of LCBs, dihydrosphingosine (DHS) and phytosphingosine (PHS), which is derived from DHS. The C-18 forms of LCBs transiently increase 2- to 3-fold, whereas C-20 forms of both DHS and PHS transiently increase 100-fold in response to heat shock (Dickson *et al.* 1997; Jenkins *et al.* 1997). DHS and PHS are phosphorylated (catalyzed by Lcb4p and Lcb5p kinases), and the level of phosphorylated LCBs can be adjusted by degradation via the Dpl1p lyase or dephosphorylated by Lcb3p and/or Ysr3p. The *lcb5Δ* mutant, that is impaired in D-erythro-sphingosine kinase activity, was very sensitive in agreement with the previous literature, and the *dpl1Δ* mutant that overaccumulates DHS-1-phosphate or PHS-1-phosphate, was very resistant. In addition, the *sur1Δ* mutant, that lacks the catalytic subunit of a mannosylinositol phosphorylceramide synthase, was very sensitive in exponential phase, indicating that mannosylated ceramides may also play a role in the maintenance of heat-shock resistance.

Phospholipid composition also appears to be important because the two mutants (*cho1Δ* and *opi3Δ*) lacking the pathway from phosphatidylethanolamine (PE) to phosphatidylcholine were very sensitive, while the *cho2Δ* mutant lacking phosphatidylserine synthase was very resistant in stationary phase, and *cho1Δ* lacking was very sensitive, possibly implicating phosphatidylethanolamine or phosphatidylserine homeostasis in resistance.

Membrane fluidity has long been known to affect ability of strains to grow at particular temperature ranges. Lockshon *et al.* (2012) have shown that mutants affected in some components of the cell wall integrity pathway, including Pkc1p, Rho1p, the Rho1p activating factor Sac7p, as well as the guanine nucleotide exchange factor Tus1p, were affected in membrane fluidity. None of these were found in the present screen, but the cell wall integrity pathway was evident in the thermotolerance data of Auesukaree *et al.* (2009), again highlighting a difference between functions that are acquired during thermotolerance that are not necessarily important for heat-shock resistance.

### Other metabolic systems affecting resistance: trehalose and riboflavin biosynthesis

Trehalose has long been implicated in heat-shock resistance and thermotolerance, and deletion of *PGM2* (encoding phosphoglucomutase) and *TPS2* (trehalose-6-phosphate phosphatase) led to sensitivity in the exponential and stationary phases, respectively (there are several trehalose-phosphate synthetases; hence, these would not be represented in the screen). All of the nonessential genes involved in riboflavin (and hence FAD and FMN) synthesis (*RIB1*, *4*, and *7*) also were represented in the stationary phase resistant set, indicating that flavins seem to have a deleterious effect after heat shock.

In summary, many genes are involved in maintaining cellular resistance to heat shock, and the genome-wide screening has identified functions known to be involved in the heat-shock response such as TOR signaling, the protein kinase A pathway and sphingolipid and trehalose metabolism, as well as a number of others that have not previously been discussed in much detail. These latter functions include the double-strand break DNA repair pathway, tryptophan metabolism, vacuolar ATPase, mitochondrial function, and ribosome biogenesis. Overall, there is a strong indication of the major role played by the TOR pathway in maintaining resistance to heat shock as has been described previously. The data indicate that oxidative stresses other than those caused by superoxide may have less impact on heat-shock resistance and point to the need for more detailed analysis of the role of oxidative stress functions in cell survival. The data also highlight the difference between thermotolerance and survival of sudden heat shock—in the former there is time for the cells to adapt to the increased temperature—which is generally lower than temperatures used to study heat-shock survival.

## Supplementary Material

Supporting Information
