# Supplementary material for: Saccharomyces cerevisiae Genes Involved in Survival of Heat Shock
Source: G3 (Bethesda). 2013 Oct 18;3(12):2321–33. doi: 10.1534/g3.113.007971 (PMC3852394; doi:10.1534/g3.113.007971)
Supplement: Supporting Information [file supp_3_12_2321__index.html]

Saccharomyces cerevisiae Genes Involved in Survival of Heat Shock — Supporting Information 

# *Saccharomyces cerevisiae* Genes Involved in Survival of Heat Shock

## Supporting Information for Jarolim *et al.*, 2013

**Files in this Data Supplement:**

- Supporting Information - Files S1-S2 and Tables S1-S2 (PDF, 518 KB)
- Table S1 - Overlap between deletion mutants affected in heat shock, and those involved in cell death as identified by Tang *et al.* (2011). (PDF, 506 KB)
- Table S2 - Percentage of genes identified in the heat-shock screens that also had >two-fold difference in expression from the Gasch *et al.* (2000) microarray data for "heat shock 20 minutes protocol hs-1." (PDF, 505 KB)
- File S1 - Data from the heat-shock screens (.xlsx, 95 KB)
- File S2 - Genes that when deleted affect heat-shock resistance or sensitivity that overlap with those whose expression is altered by heat shock (.xlsx, 16 KB)
