# Supplementary material for: Saccharomyces cerevisiae Genes Involved in Survival of Heat Shock
Source: G3 (Bethesda). 2013 Oct 18;3(12):2321–33. doi: 10.1534/g3.113.007971 (PMC3852394; doi:10.1534/g3.113.007971)
Supplement: Supporting Information [file supp_g3.113.007971_007971SI.pdf]

G3·Genes | Genomes | Genetics

Saccharomyces cerevisiae genes involved in survival of heat shock

Stefanie Jarolim[*], Anita Ayer[§], Bethany Pillay[§], Allison Gee[**], Alex Phrakaysone[**], Gabriel G. Perrone[**], Michael Breitenbach,[*] and Ian W. Dawes[§1]

[*]Division of Genetics, Department of Cell Biology, University of Salzburg, Salzburg, Austria, [§]School of Biotechnology and Biomolecular Sciences, University of New South Wales, Sydney, NSW 2052, Australia, [**]School of Science and Health, University of Western Sydney, Penrith, NSW 1797, Australia

[1]Corresponding author: School of Biotechnology and Biomolecular Sciences, UNSW, Sydney, NSW 2052, Australia. E-mail: i.dawes@unsw.edu.au

DOI: 10.1534/g3.113.007971

File S1

Data from the heat-shock screens

Gene lists are provided for non-essential genes that when deleted lead to increased or decreased resistance to 50°. Data are separately provided for cells in exponential and stationary phase. The essential gene data were obtained from the analysis of heterozygous diploids. The extent of resistance is indicated by numbers from 1 to 4 with 4 being the greatest, and for sensitivity from -1 to -4 with -4 being most sensitive.

File S1 is available for download at http://www.g3journal.org/lookup/suppl/doi:10.1534/g3.113.007971/-/DC1

File S2

Genes that when deleted affect heat-shock resistance or sensitivity that overlap with those whose expression is altered by heat shock

Heat-shock transcript data were those of Gasch *et al.*, 2000 for a heat shock protocol using addition of prewarmed medium for a shift from 25° to 37°; data from the 20 minute time sample. Summary table of percentage overlap for genes in each category is given below.

File S2 is available for download at http://www.g3journal.org/lookup/suppl/doi:10.1534/g3.113.007971/-/DC1.

Table S1   Overlap between deletion mutants affected in heat shock, and those involved in cell death as identified by Tang *et al.* (2011)

Genes listed are those identified by *Tang et al.* (2011), which when deleted lead to a similar altered resistance to heat ramp and acetic acid treatments. Those in red were not found in the heat shock resistance screen of either exponential or stationary phase cells. Overlap is 18 from 88 total genes (20% overlap)

CLN3	POM33	MMM1
SSA2	BDH2	SPO75
EMC6	FLC2	MDM10
CNE1	BPT1	SWH1
ERP2 but ERP4	SDC25	PAU8
BDH1	ERV46	YAL018C
FUN14	DRS2	FRT2
YAR023C	PEX22	AIM2
SPO7	GDH3	YAT1
YLL032C	KIN3	PSR1
UIP3	FRA1	SWD1
NTG1	FUN19	COX17 (plus COX6,16,20,23)
ENT4	SWC3	PAU7
YEH1	GEM1	TPO1
BUD14 but BUD7,19,20,23 and 31	YAL043C–a	OAF1
YAR028W	PMT2	RBG1
YLL017W	FUN26	VPS8 (plus VPS4,9,13,20,25,27,28,30,41,54,69,74)
FUN30	PUF3	
UBI4	IRC19	
KNS1	YAL065C	GIP4
SNC1	PRM9	HSP104
DNM1	RTT109	SYN8
GCV3	ATS1	YAR043C
YAR044W	SEO1	FUN12
YAL037W	SAW1	PSK1
CCR4	PAU17	ECM1
NUP60	CYC3	GPB2 but GPB1
MYO4	ADE1 but ADE5,6,8	YAR037W
AIM1	DEP1	YAR029W
SPA2	YAR040C	SSA1 but SSA2

Table S2 Percentage of genes identified in the heat-shock screens that also had >two-fold difference in expression from the Gasch *et al.* (2000) microarray data for "heat shock 20 minutes protocol hs-1".

	Exponential Sensitive	Exponential Resistant	Stationary Sensitive	Stationary Resistant
Non Essential	29.5 (93/315)	30.7 (31/101)	20.8 (42/201)	33.3 (105/314)
Essential	37.1 (13/35)	58.5 (24 /41)	38.9 (7/18)	37.5 (6/16)

Figures in parentheses indicate the total number of deletants in each category
