# Supplementary material for: Saccharomyces cerevisiae Genes Involved in Survival of Heat Shock
Source: G3 (Bethesda). 2013 Oct 18;3(12):2321–33. doi: 10.1534/g3.113.007971 (PMC3852394; doi:10.1534/g3.113.007971)
Supplement: Supporting Information [file supp_g3.113.007971_TableS1.pdf]

Table S1 Overlap between deletion mutants affected in heat shock, and those involved in cell death as identified by Tang *et al.* (2011)

Genes listed are those identified by *Tang et al.* (2011), which when deleted lead to a similar altered resistance to heat ramp and acetic acid treatments. Those in red were not found in the heat shock resistance screen of either exponential or stationary phase cells. Overlap is 18 from 88 total genes (20% overlap)

CLN3	POM33	MMM1
SSA2	BDH2	SPO75
EMC6	FLC2	MDM10
CNE1	BPT1	SWH1
ERP2 but ERP4	SDC25	PAU8
BDH1	ERV46	YAL018C
FUN14	DRS2	FRT2
YAR023C	PEX22	AIM2
SPO7	GDH3	YAT1
YLL032C	KIN3	PSR1
UIP3	FRA1	SWD1
NTG1	FUN19	COX17 (plus COX6,16,20,23)
ENT4	SWC3	PAU7
YEH1	GEM1	TPO1
BUD14 but BUD7,19,20,23 and 31	YAL043C–a	OAF1
YAR028W	PMT2	RBG1
YLL017W	FUN26	VPS8 (plus VPS4,9,13,20,25,27,28,30,41,54,69,74)
FUN30	PUF3	
UBI4	IRC19	
KNS1	YAL065C	GIP4
SNC1	PRM9	HSP104
DNM1	RTT109	SYN8
GCV3	ATS1	YAR043C
YAR044W	SEO1	FUN12
YAL037W	SAW1	PSK1
CCR4	PAU17	ECM1
NUP60	CYC3	GPB2 but GPB1
MYO4	ADE1 but ADE5,6,8	YAR037W
AIM1	DEP1	YAR029W
SPA2	YAR040C	SSA1 but SSA2
