# Supplementary material for: Saccharomyces cerevisiae Genes Involved in Survival of Heat Shock
Source: G3 (Bethesda). 2013 Oct 18;3(12):2321–33. doi: 10.1534/g3.113.007971 (PMC3852394; doi:10.1534/g3.113.007971)
Supplement: Supporting Information [file supp_g3.113.007971_TableS2.pdf]

Table S2 Percentage of genes identified in the heat-shock screens that also had >two-fold difference in expression from the Gasch *et al.* (2000) microarray data for "heat shock 20 minutes protocol hs-1".

	Exponential Sensitive	Exponential Resistant	Stationary Sensitive	Stationary Resistant
Non Essential	29.5 (93/315)	30.7 (31/101)	20.8 (42/201)	33.3 (105/314)
Essential	37.1 (13/35)	58.5 (24 /41)	38.9 (7/18)	37.5 (6/16)

Figures in parentheses indicate the total number of deletants in each category
